# Retrospective cohort study of hospital variation in airway management during in-hospital cardiac arrest and the association with patient survival: insights from Get With The Guidelines-Resuscitation

**DOI:** 10.1186/s13054-019-2426-5

**Published:** 2019-05-06

**Authors:** Steven M. Bradley, Yunshu Zhou, Satya Krishna Ramachandran, Milo Engoren, Michael Donnino, Saket Girotra

**Affiliations:** 1 Minneapolis Heart Institute and Minneapolis Heart Institute Foundation, 920 East 28th Street, Suite 300, Minneapolis, MN 55407 USA; 2 University of Iowa Carver College of Medicine, Iowa City, IA USA; 3 Beth Israel Deaconess Medical Center, Boston, MA USA; 4 University of Michigan School of Medicine, Ann Arbor, MI USA

**Keywords:** Resuscitation, Intubation, Cardiac arrest

## Abstract

**Importance:**

The optimal approach to airway management during in-hospital cardiac arrest is unknown.

**Objective:**

To describe hospital-level variation in endotracheal intubation during cardiopulmonary resuscitation (CPR) for in-hospital cardiac arrest and the association between hospital use of endotracheal intubation and arrest survival.

**Design, setting, participants:**

Retrospective cohort study of adult patients suffering in-hospital cardiac arrest at Get With The Guidelines-Resuscitation participating hospitals between January, 2000, and December, 2016. Hospitals were categorized into quartiles based on the proportion of in-hospital cardiac arrest patients managed with endotracheal intubation during CPR. Risk-adjusted mixed models with random intercepts were created to assess the association between hospital quartile of in-hospital arrests managed with endotracheal intubation during CPR and survival to hospital discharge.

**Exposure:**

Hospital rate of endotracheal intubation during CPR for in-hospital arrest

**Main outcomes and measures:**

Survival to hospital discharge

**Results:**

Among 155,252 patients suffering in-hospital cardiac arrest at 656 hospitals, 69.7% of patients received endotracheal intubation during CPR and overall survival to discharge was 24.8%. At the hospital level, the median rate of endotracheal intubation use was 71.2% (interquartile range, 63.6 to 78.1%; range, 26.6 to 100%). We found a strong inverse association between hospital rate of endotracheal intubation and survival to discharge (risk-adjusted odds ratio comparing highest intubation quartile vs. lowest intubation quartile, 0.81; 95% confidence interval (CI), 0.74 to 0.90; *p* value < .001). This association was modified by the presence of respiratory failure prior to arrest (*p* for interaction < .001), and stratified analyses demonstrated lower patient survival at hospitals with higher rates of endotracheal intubation was limited to patients without respiratory failure prior to cardiac arrest.

**Conclusion:**

In a national sample of patients suffering IHCA, the use of endotracheal intubation during CPR varied across hospitals. We found a strong inverse association between hospital use of endotracheal intubation during CPR and survival to discharge, but this association was confined to patients without respiratory failure prior to arrest. Identifying the optimal approach to airway management for in-hospital cardiac arrest may have a significant impact on patient survival.

## Background

More than 200,000 patients suffer in-hospital cardiac arrest (IHCA) annually in the USA with an in-hospital mortality that approaches 80% [1, 2]. Airway management is a central component of resuscitation care, but it is unclear if endotracheal intubation improves patient survival. As the process of tracheal intubation often requires cessation of chest compressions [3, 4] and may result in delays in timely defibrillation or epinephrine administration [5, 6], avoiding intubation may minimize interruptions in aspects of high-quality resuscitation care [7]. In fact, studies from both the in- and out-of-hospital setting have associated resuscitation strategies that delay or minimize intubation with improved patient survival [8–12]. Resuscitation guidelines now support either invasive or non-invasive approaches to ventilation and oxygenation in the management of cardiac arrest [13].

Although prior patient-level studies of IHCA suggest a negative association between endotracheal intubation and patient survival [12], less is known about hospital practices in airway management during CPR and the association without outcomes. Given clinical uncertainty about the optimal approach to airway management in resuscitation care, variation may exist in the use of tracheal intubation during cardiac arrest. Evaluating rates of hospital use of intubation during cardiac arrest and the association with patient outcomes may provide insights on resuscitation practices with the potential to improve patient survival.

Accordingly, we analyzed data from the Get With The Guidelines-Resuscitation (GWTG-R) registry to describe hospital rates of endotracheal intubation during IHCA and evaluated the association between hospital rates of intubation and survival outcomes. We hypothesized that hospitals with lower rates of intubation during IHCA would be associated with greater survival. Prior studies suggest the association between intubation and arrest outcomes may be modified by the presence of respiratory failure and arrest rhythm, with greater potential harm of intubation with ventricular tachycardia or ventricular fibrillation (VT/VF) arrest or non-respiratory arrest. Accordingly, we also assessed for modification of the association between airway management and patient outcomes by initial arrest rhythm and the presence or absence of respiratory failure. We hypothesized that the inverse association between hospital rates of intubation and patient outcomes would be stronger for VT/VF arrests and non-respiratory arrests.

## Methods

### Data source

We analyzed data from the GWGT-R^8^ registry, an American Heart Association sponsored prospective, multi-site registry of in-hospital cardiac arrest events. The GWTG-R has been described previously in detail [9]. Briefly, an IHCA event is defined in the registry as a pulseless cardiac arrest that requires chest compressions and/or defibrillation. Data abstraction for each IHCA is performed by trained personnel at each participating institution [8]. Data integrity and accuracy is ensured through use of standardized reporting software and certification of data entry personnel [10].

### Patient population

We included patients aged 18 years or older who experienced IHCA at a GWTG-R participating hospital from January 1, 2000, to December 31, 2016. If a patient had multiple IHCAs, we excluded data from subsequent episodes to focus on the index event. Patients with pre-existing invasive ventilation prior to the arrest were excluded from the analysis. We also excluded patients with missing data on airway management, first pulseless rhythm, or survival outcomes. As our analyses were conducted at the hospital level, we excluded hospitals with fewer than 10 arrests to avoid inflation of variation due to small sample sizes.

### Primary exposure

We identified patients who underwent placement of an endotracheal tube during CPR. Use of a bag valve mask, nasal mask, mouth-to-mouth ventilation, or a laryngeal mask airway during CPR was considered non-tracheal intubation approaches to airway management and not included in the exposure group.

### Study outcomes

The primary outcome measure was survival to hospital discharge. The secondary outcomes included return of spontaneous circulation and survival to 24 h.

### Statistical analysis

We determined the proportion of patients treated with endotracheal intubation during CPR at GWTG-R participating hospitals. We then categorized hospitals into quartiles based on the proportion of patients who received tracheal intubation during CPR. We compared patient and hospital-level characteristics across hospital quartile of intubation during CPR using Cochrane-Armitage test for categorical variables and simple linear regression for continuous variables.

We next constructed a two-level hierarchical multivariable model to evaluate the risk-adjusted association between the hospital quartile of intubation during CPR and our primary and secondary outcomes. In these models, hospital site was added as a random effect and we adjusted for patient-level variables and hospital quartile of endotracheal intubation as fixed effects. Patient-level covariates for risk adjustment were chosen from previously validated survival models [11] using the GWTG-R database and included age, sex, race category (white, black, other), initial arrest rhythm (asystole, pulseless electrical activity, ventricular fibrillation, and ventricular tachycardia), hospital location of arrest (intensive care unit, monitored ward, non-monitored ward, procedural area/emergency department, other), witnessed arrest, time of arrest (daytime [7 A.M. to 10:59 P.M.], night [11 P.M. to 6:59 A.M.]), day of the week (weekday [Monday–Friday], weekend [Saturday or Sunday]), use of a hospital-wide code alert, pre-existing medical conditions (heart failure during current or prior admission, myocardial infarction during current or prior admission, hypotension, respiratory failure, renal insufficiency, hepatic insufficiency, metabolic or electrolyte abnormality, diabetes mellitus, baseline depression in central nervous system function, acute stroke, pneumonia septicemia, major trauma, and cancer), and interventions in place at prior to the arrest (use of intravenous vasoactive agents, intra-arterial catheter, and dialysis).

We evaluated the association between quartiles of hospital intubation rates and study outcomes using hierarchical logistic regression models with hospital-specific random intercepts. We also examined for effect modification (interaction) of the association between hospital intubation rates and patient outcomes by initial arrest rhythm and the presence or absence of respiratory failure. Further stratified analyses were planned if the interaction term was significantly associated with survival to discharge, stratified by presence or absence of ventricular tachycardia or ventricular fibrillation as the initial presenting rhythm and presence or absence of respiratory failure prior to arrest. For all analyses, the null hypothesis was evaluated at a two-sided significance level of 0.05. SAS Software Version 9.4 (SAS Institute Inc., Cary, NC).

## Results

**Fig. 1:**
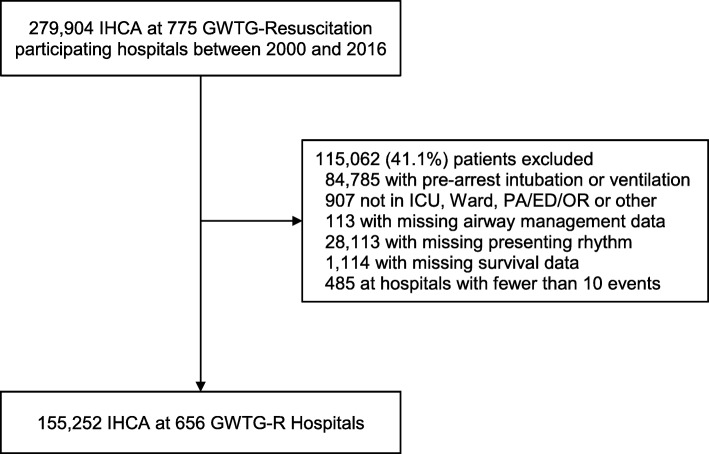
Cohort identification

**Fig. 2:**
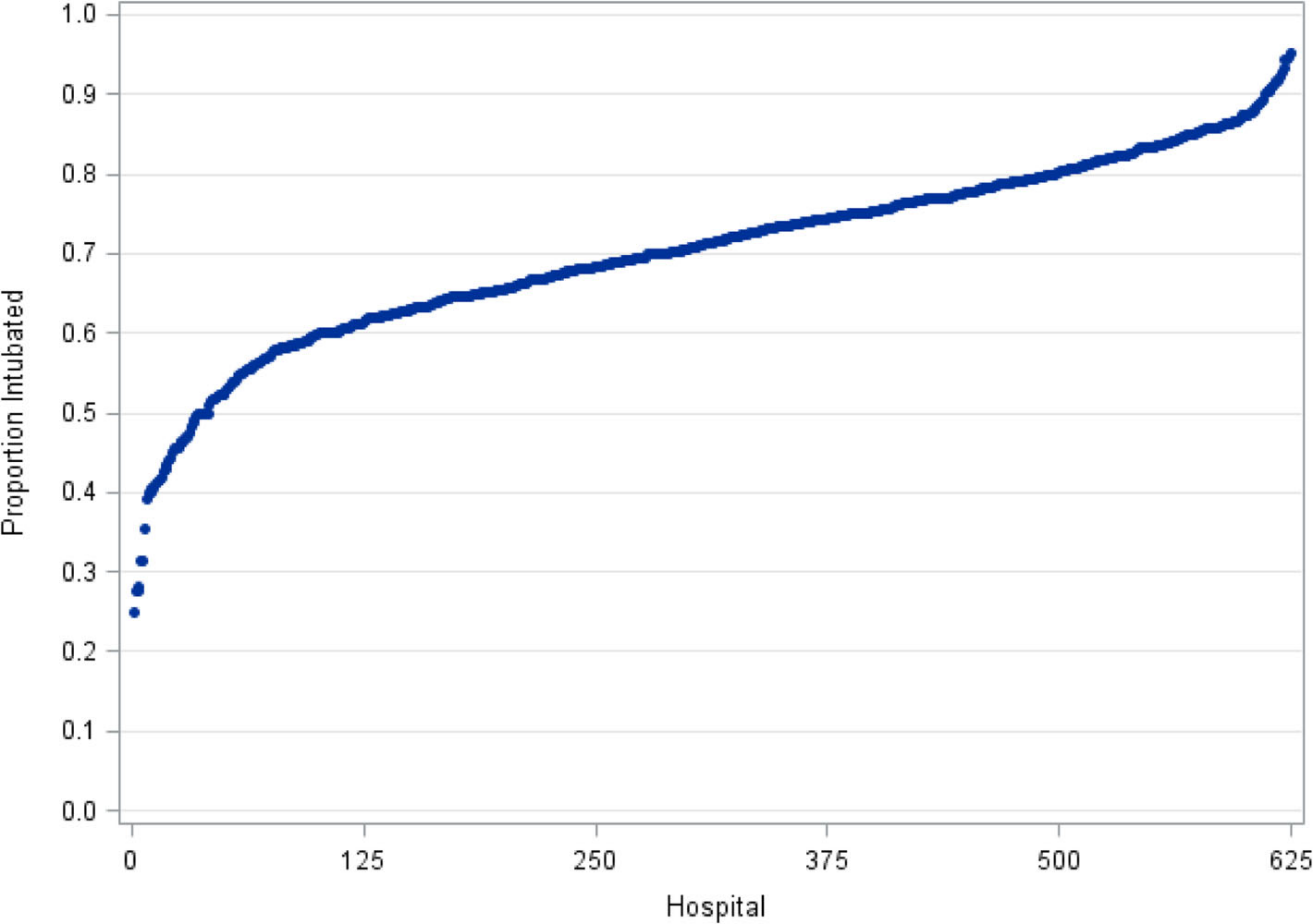
Hospital variation in the proportion of patients intubated during CPR

We identified 155,252 index IHCAs at 656 hospitals (Fig. 1). Endotracheal intubation was used during CPR in 108,221 (69.7%) patients overall, 17,422 (55.1%) patients with an initial presenting rhythm of VT/VF, 90,799 (73.5%) patients with PEA or asystolic arrests, 35,850 (68.4%) patients with respiratory failure, and 72,371 (70.4%) patients without respiratory failure. There was substantial variation in hospital intubation rates during CPR, with a median of 71.2% and a range of 26.6 to 100.0% (Fig. 2).

Table 1 displays the baseline differences among patients across the intubation rate quartile hospitals. Compared with patients at hospitals in the lowest quartile of intubation rates, patients at hospitals in the highest quartile of intubation were more likely to be black (24.8% vs 17.5%), arrest in a non-monitored unit (27.2% vs 19.8%), and have a hospital-wide code activated (80.8% vs 74.2%). Other statistically significant but clinically small differences were noted among many characteristics given the large size of the study cohort.

**Table 1:** Patient and hospital characteristics by hospital quartiles of endotracheal intubation during CPR

Patient characteristics	All patients *N* = 155,252	1—lowest (*n* = 32,825)	2 (*n* = 44,535)	3 (*n* = 47,480)	4—highest (*n* = 30,412)	*P* value for trend
Median intubation rate during CPR, % (IQR)	71.4 (64.7, 76.7)	57.6 (50.0, 61.3)	67.5 (65.6, 69.0)	75.0 (72.8, 76.4)	80.4 (79.5, 83.1)	
Patient demographics						
Age, mean (SD)	67.17 (15.3)	66.01 (15.5)	67.02 (15.3)	67.69 (15.1)	67.85 (15.3)	< .001
Black race	32,044 (20.6)	5752 (17.5)	8721 (19.6)	10,032 (21.1)	7539 (24.8)	< .001
Female sex	64,369 (41.5)	13,342 (40.7)	18,576 (41.7)	19,516 (41.1)	12,935 (42.5)	< .001
Cardiac arrest characteristics						
Initial cardiac-arrest rhythm—no. (%)						< .001
Asystole	52,250 (33.7)	10,088 (30.7)	14,887 (33.4)	16,231 (34.2)	11,044 (36.3)	
Pulseless electrical activity	71,370 (46.0)	15,437 (47.0)	20,283 (45.5)	21,891 (46.1)	13,759 (45.2)	
Pulseless ventricular tachycardia	11,512 (7.4)	2705 (8.2)	3463 (7.8)	3345 (7.1)	1999 (6.6)	
Ventricular fibrillation	20,120 (13.0)	4595 (14.0)	5902 (13.3)	6013 (12.7)	3610 (11.9)	
Hospital location of arrest—no. (%)						< .001
Intensive care unit	53,274 (34.3)	12,494 (38.1)	16,269 (36.5)	15,463 (32.6)	9048 (30.0)	
Monitored unit	35,249 (22.7)	6513 (19.8)	9691 (21.8)	11,476 (24.2)	7569 (24.9)	
Non-monitored unit	35,611 (22.9)	6500 (19.8)	9685 (21.8)	11,170 (23.5)	8256 (27.2)	
Procedural areas/OR/ED	28,749 (18.5)	6914 (21.1)	8231 (18.5)	8530 (18.0)	5074 (16.7)	
Other	2369 (1.5)	404 (1.2)	659 (1.5)	841 (1.8)	465 (1.5)	
Arrest at night (11 P.M. to 7 A.M.)—no./total no. (%)	49,611 (32.0)	10,104 (30.8)	14,161 (31.8)	15,401 (32.4)	9945 (32.7)	< .001
Arrest on weekend—no. (%)	47,933 (30.9)	10,104 (30.8)	13,713 (30.8)	14,746 (31.1)	9370 (30.8)	0.68
Hospital-wide response activated—no. (%)	125,741 (81.0)	24,342 (74.2)	36,713 (82.4)	40,103 (84.5)	24,583 (80.8)	< .001
Preexisting conditions						
Heart failure, this admission—no. (%)	26,227 (16.9)	5185 (15.8)	6717 (15.1)	8345 (17.6)	5980 (19.7)	< .001
Previous heart failure—no. (%)	35,040 (22.6)	7322 (22.3)	9657 (21.7)	10,454 (22.0)	7607 (25.0)	< .001
Myocardial infarction, this admission—no. (%)	25,132 (16.2)	5312 (16.2)	6979 (15.7)	7516 (15.8)	5325 (17.5)	< .001
Previous myocardial infarction—no. (%)	25,137 (16.2)	4948 (15.1)	6561 (14.7)	7533 (15.9)	6095 (20.0)	< .001
Hypotension—no. (%)	30,309 (19.5)	6913 (21.1)	7985 (17.9)	9030 (19.0)	6381 (21.0)	0.37
Respiratory failure—no. (%)	52,430 (33.8)	11,011 (33.5)	14,960 (33.6)	15,938 (33.6)	10,521 (34.6)	0.01
Renal insufficiency—no. (%)	51,151 (33.0)	10,037 (30.6)	14,588 (32.8)	15,707 (33.1)	10,819 (35.6)	< .001
Hepatic insufficiency—no. (%)	9913 (6.4)	1901 (5.8)	2822 (6.3)	2922 (6.2)	2268 (7.5)	< .001
Metabolic or electrolyte abnormality—no. (%)	22,673 (14.6)	5119 (15.6)	6015 (13.5)	6483 (13.6)	5056 (16.6)	0.002
Diabetes mellitus—no. (%)	50,550 (32.6)	10,431 (31.8)	14,159 (31.8)	15,547 (32.7)	10,413 (34.2)	< .001
Baseline depression in CNS function—no. (%)	15,097 (9.7)	2954 (9.0)	3841 (8.6)	4640 (9.8)	3662 (12.0)	< .001
Acute stroke—no. (%)	5812 (3.7)	1260 (3.8)	1614 (3.6)	1724 (3.6)	1214 (4.0)	0.39
Pneumonia—no. (%)	18,529 (11.9)	3971 (12.1)	5063 (11.4)	5788 (12.2)	3707 (12.2)	0.09
Septicemia—no. (%)	20,934 (13.5)	4344 (13.3)	5849 (13.1)	6253 (13.2)	4488 (14.8)	< .001
Major trauma—no. (%)	4125 (2.7)	1348 (4.1)	1076 (2.4)	1064 (2.2)	637 (2.1)	< .001
Metastatic cancer—no. (%)	18,557 (12.0)	3478 (10.6)	5086 (11.4)	5873 (12.4)	4120 (13.6)	< .001
Interventions in place before the arrest						
Intravenous vasopressor medication—no. (%)	21,813 (14.1)	5587 (17.0)	6611 (14.8)	6030 (12.7)	3585 (11.8)	< .001
Dialysis—no. (%)	3823 (2.5)	783 (2.4)	1094 (2.5)	1010 (2.1)	936 (3.1)	< .001
Intra-arterial catheter	6652 (4.3)	1958 (6.0)	1966 (4.4)	1493 (3.1)	1235 (4.1)	< .001
Witnessed	122,016 (78.6)	26,509 (80.8)	35,904 (80.6)	36,711 (77.3)	22,892 (75.3)	< .001
Hospital characteristics	All hospitals *N* = 656	1—lowest (*n* = 165)	2 (*n* = 164)	3 (*n* = 163)	4—highest (*n* = 164)	*P* value for trend
Geographic census region, number (%)						0.66
Northeast	101 (15.4)	26 (15.8)	29 (17.7)	22 (13.5)	24 (14.6)	
Midwest	134 (20.4)	36 (21.8)	37 (22.6)	28 (17.2)	33 (20.1)	
West	111 (16.9)	43 (26.1)	30 (18.3)	28 (17.2)	10 (6.1)	
South	264 (40.2)	39 (23.6)	60 (36.6)	81 (49.7)	84 (51.2)	
Unknown	46 (7.0)	21 (12.7)	8 (4.9)	4 (2.5)	13 (7.9)	
Type of hospital, number (%)						0.95
Teaching hospital	319 (48.6)	81 (49.1)	85 (51.8)	78 (47.9)	75 (45.7)	
Non-teaching hospital	286 (43.6)	62 (37.6)	71 (43.3)	80 (49.1)	73 (44.5)	
Unknown	51 (7.8)	22 (13.3)	8 (4.9)	5 (3.1)	16 (9.8)	
Location, number (%)						0.21
Urban	538 (82.0)	125 (75.8)	142 (86.6)	139 (85.3)	132 (80.5)	
Rural	67 (10.2)	18 (10.9)	14 (8.5)	19 (11.7)	16 (9.8)	
Unknown	51 (7.8)	22 (13.3)	8 (4.9)	5 (3.1)	16 (9.8)	
Ownership						0.60
Private	99 (15.1)	20 (12.1)	22 (13.4)	26 (16.0)	31 (18.9)	
Public	511 (77.9)	124 (75.2)	134 (81.7)	133 (81.6)	120 (73.2)	
Unknown	46 (7.0)	21 (12.7)	8 (4.9)	4 (2.5)	13 (7.9)	
Bed size, number (%)						0.35
< 100	57 (8.7)	22 (13.3)	9 (5.5)	13 (8.0)	13 (7.9)	
100–499	414 (63.1)	92 (55.8)	114 (69.5)	109 (66.9)	99 (60.4)	
≥ 500	130 (19.8)	30 (18.2)	30 (18.3)	33 (20.3)	37 (22.6)	
Unknown	55 (8.4)	21 (12.7)	11 (6.7)	8 (4.9)	15 (9.2)	

*Abbreviations*: *CPR* cardiopulmonary resuscitation, *IQR* interquartile range, *SD* standard deviation, *OR* operating room, *ED* emergency department, *no*. number

A total of 38,435 (24.8%) of patients survived to hospital discharge. The unadjusted discharge survival decreased from 27.2% in the hospital quartile with the lowest intubation rate to 22.4% in the hospital quartile with the highest intubation rate (Table 2). Compared with hospitals in the lowest quartile of intubation use, risk-adjusted survival to discharge was lower in all quartiles with higher intubation use and lowest in the hospital quartile with highest intubation use (odds ratio (OR), 0.81; 95% confidence interval (CI), 0.74 to 0.90, *p* ≤ .0001). Similar trends were observed for our secondary outcomes of ROSC and survival to 24 h (Table 2).

**Table 2:** Survival outcomes by hospital quartile of endotracheal intubation during CPR

	All patients	Hospital quartile of intubation rate during CPR			
		1 (lowest)	2	3	4 (highest)
Survival to discharge					
Unadjusted rate, *N* (%)	38,435 (24.8)	8926 (27.2)	11,432 (25.7)	11,265 (23.7)	6812 (22.4)
Adjusted odds ratio, (95% CI)		Reference	0.95 (0.86, 1.04) *P* = 0.26	0.86 (0.78, 0.94) *P* = .002	0.81 (0.74, 0.90) *P* < .001
ROSC					
Unadjusted rate, *N* (%)	99,881 (64.4)	22,283 (67.9)	28,657 (64.4)	29,910 (63.0)	19,031 (62.6)
Adjusted odds ratio, (95% CI)		Reference	0.91 (0.81, 1.01) *P* = 0.08	0.84 (0.75, 0.94) *P* = 0.002	0.83 (0.74, 0.93) *P* = .001
Survival to 24 h					
Unadjusted rate, *N* (%)	65,739 (42.3)	14,419 (43.9)	19,066 (42.8)	19,792 (41.7)	12,462 (41.0)
Adjusted odds ratio, (95% CI)		Reference	0.96 (0.88, 1.04) *P* = 0.26	0.89 (0.82, 0.97) *P* = 0.006	0.87 (0.80, 0.95) *P* = 0.001

*Abbreviations*: *N* number, *CI* confidence interval, *CPR* cardiopulmonary resuscitation

Overall rates of intubation differed by the presence or absence of respiratory failure (68.4% with respiratory failure present, 70.4% when respiratory failure absent; *p* < .0001) and presenting arrest rhythm (55.1% with VT/VF, 73.5% with asystole/PEA; *p* < .0001). The association between hospital endotracheal intubation use and outcomes was modified by the presence of respiratory failure (*P* for interaction < .0001 for all outcomes) but not presenting rhythm (*P* = 0.30). Stratified on the absence or presence of respiratory failure, patients without respiratory failure were more likely to survive IHCA at hospitals with low rates of tracheal intubation use, but no association was observed between hospital rates of tracheal intubation use and survival of IHCA among patients with respiratory failure (Table 3). The inverse association between hospital intubation rates and survival was observed for both VT/VF and PEA/asystolic arrests (Table 4).

**Table 3:** Survival outcomes by hospital quartile of endotracheal intubation during CPR stratified by the presence or absence of respiratory failure

	All patients	Hospital quartile of intubation rate during CPR			
		1 (lowest)	2	3	4 (highest)
Respiratory failure present					
Survival to discharge					
Unadjusted rate, *N* (%)	11,374 (21.6)	2387 (21.7)	3407 (22.8)	3351 (21.0)	2229 (21.2)
Adjusted odds ratio (95% CI)		Reference	1.07 (0.95, 1.19) P = 0.26	0.95 (0.85, 1.06) *P* = 0.38	0.97 (0.86, 1.09) *P* = 0.61
ROSC					
Unadjusted rate, *N* (%)	34,700 (66.2)	7515 (68.3)	9983 (66.7)	10,354 (65.0)	6848 (65.1)
Adjusted odds ratio (95% CI)		Reference	0.99 (0.87, 1.11) *P* = 0.81	0.89 (0.79, 1.00) *P* = .06	0.86 (0.76, 0.98) *P* = 0.02
Survival to 24 h					
Unadjusted rate, *N* (%)	22,101 (42.2)	4497 (40.8)	6430 (43.0)	6683 (41.9)	4491 (42.7)
Adjusted odds ratio (95% CI)		Reference	1.07 (0.97, 1.17) *P* = 0.17	1.01 (0.92, 1.11) *P* = 0.88	1.01 (0.91, 1.11) *P* = 0.87
Respiratory failure absent					
Survival to discharge					
Unadjusted Rate, *N* (%)	27,061 (26.3)	6539 (30.0)	8025 (27.1)	7914 (25.1)	4583 (23.0)
Adjusted odds ratio (95% CI)		Reference	0.92 (0.83, 1.02) *P* = 0.10	0.83 (0.75, 0.92) *P* < .001	0.76 (0.68, 0.85) *P* < .001
ROSC					
Unadjusted rate, *N* (%)	65,181 (63.4)	14,768 (67.7)	18,674 (63.2)	19,556 (62.0)	12,183 (61.3)
Adjusted odds ratio (95% CI)		Reference	0.87 (0.78, 0.97) *P* = .01	0.82 (0.73, 0.91) *P* < .001	0.81 (0.72, 0.90) *P* < .001
Survival to 24 h					
Unadjusted rate, *N* (%)	43,638 (42.4)	9922 (45.5)	12,636 (42.7)	13,109 (41.6)	7971 (40.1)
Adjusted odds ratio (95% CI)		Reference	0.91 (0.84, 1.00) *P* = 0.04	0.86 (0.79, 0.94) *P* = 0.001	0.82 (0.75, 0.90) *P* < .001

*Abbreviations*: *N* number, *CI* confidence interval, *CPR* cardiopulmonary resuscitation

**Table 4:** Survival outcomes by hospital quartile of endotracheal intubation during CPR and stratified by presenting rhythm

	All patients	Hospital quartile of intubation rate during CPR			
		1 (lowest)	2	3	4 (highest)
VT/VF					
Survival to discharge					
Unadjusted rate, *N* (%)	14,898 (47.1)	3677 (50.4)	4436 (47.4)	4345 (46.4)	2440 (43.5)
Adjusted odds ratio (95% CI)		Reference	0.94 (0.83, 1.06) *P* = 0.29	0.88 (0.78, 0.99) *P* = 0.03	0.82 (0.72, 0.93) *P* = 0.002
ROSC					
Unadjusted rate, *N* (%)	24,040 (76.0)	5833 (80.0)	7063 (75.5)	7036 (75.2)	4108 (73.3)
Adjusted odds ratio, (95% CI)		Reference	0.82 (0.72, 0.94) *P* = 0.006	0.78 (0.68, 0.90) *P* = 0.001	0.70 (0.60, 0.81) *P* < .001
Survival to 24 h					
Unadjusted rate, *N* (%)	19,651 (62.1)	4716 (64.6)	5823 (62.2)	5785 (61.8)	3327 (59.3)
Adjusted odds ratio (95% CI)		Reference	0.94 (0.84, 1.05) *P* = 0.26	0.90 (0.80, 1.00) *P* = 0.05	0.80 (0.71, 0.91) *P* = 0.001
PEA/asystole					
Survival to discharge					
Unadjusted rate, *N* (%)	23,537 (19.0)	5249 (20.6)	6996 (19.9)	6920 (18.2)	4372 (17.6)
Adjusted odds ratio (95% CI)		Reference	0.95 (0.85, 1.05) *P* = 0.30	0.84 (0.76, 0.94) *P* = 0.002	0.82 (0.73, 0.91) *P* < .001
ROSC					
Unadjusted rate, *N* (%)	75,841 (61.4)	16,450 (64.5)	21,594 (61.4)	22,874 (60.0)	14,923 (60.2)
Adjusted odds ratio (95% CI)		Reference	0.92 (0.82, 1.03) *P* = 0.16	0.84 (0.75, 0.94) *P* = 0.003	0.85 (0.76, 0.96) *P* = 0.008
Survival to 24 h					
Unadjusted rate, *N* (%)	46,088 (37.3)	9703 (38.0)	13,243 (37.7)	14,007 (36.7)	9135 (36.8)
Adjusted odds ratio (95% CI)		Reference	0.96 (0.88, 1.04) *P* = 0.33	0.90 (0.82, 0.97) *P* = 0.01	0.90 (0.82, 0.98) *P* = 0.02

*Abbreviations*: *N* number, *CI* confidence interval, *CPR* cardiopulmonary resuscitation

## Discussion

In a national registry of more than 150,000 IHCA and more than 650 hospitals, we described variation in the use of endotracheal intubation during resuscitation efforts and the association between hospital rates of tracheal intubation use and patient outcomes. Use of endotracheal intubation was common with 70% of patients receiving a tracheal intubation during CPR. However, hospital rates of tracheal intubation use varied from 27 to 100%. Compared to hospitals with more frequent use of tracheal intubation, hospitals with lower rates of tracheal intubation use were associated with better patient survival. This association was modified by the presence or absence of respiratory failure, with the association between lower rates of tracheal intubation use and better survival being limited to patients without respiratory failure. These findings are consistent with prior patient-level analyses and highlight the importance of further investigation to define the optimal approach to tracheal intubation use in the management of patients suffering in-hospital arrest.

Prior studies of cardiac arrest have suggested the potential for reduced use of intubation during CPR to improve patient outcomes [8–10, 12]. For example, in several pre-post studies of a resuscitation strategy that included non-tracheal intubation management, an approach that minimized use of intubation was associated with improved survival in out-of-hospital cardiac arrest [8–10]. However, several modifications were made to the CPR algorithm in these studies and the relative contribution of tracheal intubation use on patient outcomes was unclear. Subsequently, a propensity-matched analysis from GWTG-R demonstrated intubation within the first 15 min of CPR compared with no intubation to be associated with lower survival [12]. However, while this prior analysis adjusted for confounding due to indication by matching patients who underwent endotracheal intubation at a given minute during resuscitation with control patients who had received CPR for the same duration, potential for unmeasured confounding still remains [14]. Therefore, our findings of a strong association between rates of endotracheal intubation during in-hospital cardiac arrest and survival at a hospital-level lends further support to the findings of prior patient-level studies.

Use of tracheal intubation during resuscitation efforts has several mechanisms by which it could contribute to patient outcomes [15]. Attempts at tracheal intubation may result in delays to timely defibrillation [5], epinephrine administration [6], or interruption of chest compressions [16]. Studies of pre-hospital CPR have noted tracheal intubation placement is associated with a median 46-s interruption in chest compressions and nearly a third of interruptions exceeded 1 min [3]. Endotracheal intubation may also facilitate greater ease of ventilation and thereby potentiate excessive ventilation and oxygenation, both contributors to poor patient outcomes [17, 18].

Despite these potential drawbacks, it is important to recognize scenarios where use of tracheal intubation may have particular benefit. Animal model studies have suggested the importance of ventilation in scenarios that simulate respiratory arrest [19]. Accordingly, several pre-post studies evaluating delayed use of tracheal intubation have limited this change in practice to patients with arrest of presumed cardiac origin and a shockable arrest rhythm [8, 9]. In a prior observational study of IHCA from GWTG-R, intubation was not associated with worse survival among patients with preceding respiratory failure [12]. As such, the relative importance of tracheal intubation use to optimize patient outcomes may depend on the arrest etiology and presenting rhythm. These prior studies informed our decision to evaluate for effect modification on initial arrest rhythm and the presence or absence of respiratory failure. Our study findings further support a differential importance of tracheal intubation in resuscitation care depending on arrest etiology, with the inverse association between intubation use and patient outcomes being strongest in patients without respiratory failure prior to arrest. Survival outcomes in patients with respiratory insufficiency prior to arrest were similar across hospital intubation quartiles. These findings also highlight that our exposure is not a surrogate for resuscitation quality, as patient outcomes were not uniformly better for all patient populations at hospitals with lower intubation rates.

Randomized trials of invasive vs non-invasive intubation management for out-of-hospital cardiac arrest are ongoing [20]. However, significant differences in the arrest characteristic and management of in-hospital cardiac arrest present challenges in applying the findings from these trials to the inpatient setting. Unlike out-of-hospital cardiac arrest where half of events are unwitnessed and CPR is often delayed [21], the vast majority of in-hospital cardiac arrests occur in monitored settings with trained personnel to facilitate resuscitation care and minimal time to onset of resuscitation efforts [22]. These differences may influence the relative importance of endotracheal intubation during CPR. As such, further study focusing on the optimal approach to airway management of in-hospital arrest is needed.

Our study findings should be interpreted in the context of several limitations. First, GWTG-R participating hospitals may not be representative of all US hospitals and therefore our findings may not be generalizable. Second, the GWTG-R does not collect data on the reasons for specific airway choices, the number of airway insertion attempts, and measures of difficult laryngoscopy or intubation that may have implications for the analysis. Third, use of laryngeal mask airways was low (< 1%) such that subgroup analysis on different types of airways was not feasible. Fourth, we cannot exclude the potential for resuscitation time bias in which an exposure is more likely to occur the longer the cardiac arrest continues [23]. However, this concern is lessened a prior study that demonstrated higher patient survival at hospitals with longer median duration of resuscitation events [24]. Fifth, given the observational nature of our study, there is potential for confounding, although we performed robust risk adjustment to address this concern. Despite these limitations, our study provides insights into current practice and the potential importance of airway management protocols on patient survival.

## Conclusions

Among more than 150,000 patients suffering in-hospital cardiac arrest at more than 650 hospitals, the rate of tracheal intubation use during resuscitation efforts varied from 27 to 100%. Hospitals with less frequent use of tracheal intubations during CPR were associated with better patient survival. This above association was modified by the presence or absence of respiratory failure with the apparent benefit of lower hospital rates of tracheal intubation use being limited to patients without respiratory failure. A better understanding of optimal airway management in the care of patients suffering in-hospital cardiac arrest is needed.
